# Agness Mseteka: Maternity Waiting Home Caretaker and Protector of Pregnant Women in Rural Zambia

**DOI:** 10.5334/aogh.3253

**Published:** 2021-06-25

**Authors:** Julie M. Buser, Brenda Moyo

**Affiliations:** 1 University of Michigan, 1540 E Hospital Dr, Ann Arbor, MI 48109, US; 2 Research Assistant, Lundazi, Zambia

## Abstract

To share her story, we interviewed Agness Mseteka about her experience as a maternity waiting home (MWH) caretaker at the district hospital in rural Lundazi, Zambia. Maternity waiting homes, also known as mother’s shelters, are structures built near healthcare facilities to minimize the critical barrier of distance to accessing maternal health services. Agness’ story highlights the central role caretakers play in contributing to the successful implementation and sustainability of MWH interventions. Agness is well positioned to be an agent of positive change by bringing health education to pregnant women. An important lesson learned from Agness’ story is the need for future research to explore the sustainability of MWHs and long-term effectiveness of income generating activities by the community after the completion of externally funded implementing programs.

Agness Mseteka didn’t expect to be caretaker of the maternity waiting home (MWH) at the district hospital in rural Lundazi, Zambia. Maternity waiting homes, also known as mother’s shelters, are structures built near healthcare facilities to minimize the critical barrier of distance to accessing maternal health services [1]. Agness was born in 1969 in Luanshya in the Copperbelt province of Zambia. Her father died when she was very young. She was educated at home in her mother’s village by her older sister. Eventually, Agness had four children, raising them as a single mother after divorcing her husband. She worked on a cooperative farm for almost eight years then transitioned to doing odd jobs in the village to provide for her children. To share her story, we interviewed Agness about her experience as a MWH caretaker in a combination of English and the local languages of Njanja and Tumbuka, then transcribed the recording into English. ***Figure 1*** shows a photo of Alice.

**Figure 1:**
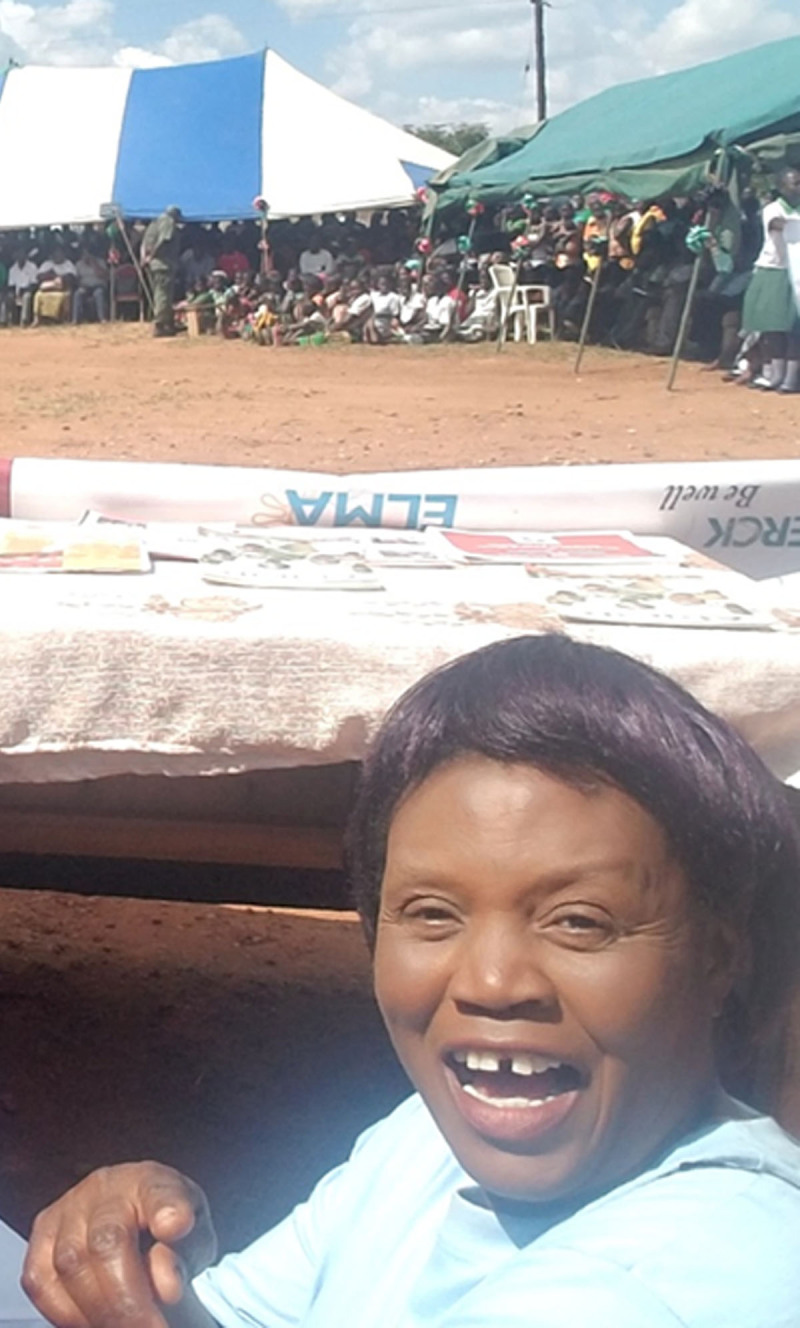
Caretaker Agness Mseteka at a government celebration of maternal-newborn health in Lundazi, Eastern Province, Zambia.

Agness became interested in health related issues after multiple conversations with a friend who worked in a medical clinic. One day, they went to the clinic where Agness helped her friend by recording the weight of pediatric patients in their charts. She really enjoyed helping at the clinic. After moving to Lundazi, she started volunteering at the largest Ministry of Health clinic in town. She provided education to mothers about pregnancy and how to take care of babies. She did this voluntary work for a number of years.

While volunteering at the clinic, she heard about a workshop to train community health workers focused on maternal-newborn health. The workshop was associated with an intervention to determine the impact of MWHs on facility delivery among women living at least 10 km from health facilities in rural Zambia [2]. Maternity waiting homes need a caretaker to manage and execute day-to-day operations. ***Table 1*** highlights some of the responsibilities for the MWH caretaker. The position is accountable to the MWH Committee. The purpose of the community-elected committee is to oversee the staff, operations and finances of the MWH and the associated income generating activities to cover costs of running the MWH. Agness enthusiastically participated in the training and to her surprise, at the end of the workshop, community health workers elected Agness as caretaker of the Lundazi District Hospital MWH.

**Table 1:** Maternity waiting home caretaker responsibilities.


Collect and maintain records of all pregnant women registered in the MWH.

Orient pregnant women and caregivers on the MWH facilities.

Communicate MWH rules, expectations and procedures.

Ensure that the needs of all pregnant women staying at the MWH are met.

Oversee the creation, maintenance and execution of a training and education schedule.

Ensure the overall security of the MWH and its occupants.

Collect MWH user contribution.

Manage order and purchase of MWH supplies.

Maintain regular inventory of all MWH assets, properties and supplies.


During her tenure as caretaker, Agness developed in-depth knowledge about pregnancy care. She enjoys educating mothers who come from various rural areas. She explained:

Being a caretaker has got merits and demerits for I feel it takes a lot to understand different situations and handle them patiently. Before, I knew mainly about hygiene, dressing, and caring of newborn babies. Through workshops I learned details about nutrition, what a pregnant mother can eat to have a healthy baby, and how mothers should start their antenatal visits as early as possible to allow them to have good health during and after their pregnancy.

Sometimes Agness feels stress in her role as caretaker of the MWH, expressing contextually and culturally specific challenges she faces. She elaborated:

After my training, I understand not to use traditional herbs to induce labor. When the mothers check-in to the MWH, I look in their bags to make sure they didn’t carry any traditional medicine which can affect their delivery. If they drink the traditional herbs—we call it *lupusu*—they think they will deliver fast without much pain. In fact, the use of our herbs was causing many mothers to die before actual labor started.

Another stress for Agness is that she occasionally faces disrespect from Ministry of Health staff working at the neighboring hospital. She mentioned:

Nurses’ attitude at times brings my morale low because they become harsh to the mothers for nothing. When a mother is not feeling too well, I take the patient to the maternity ward. At the reception, we will sometimes be given a rough time by nurses depending on the staff I have found there. For example, one time I took a mother to the hospital and she was told to go back to the mother’s shelter but she ended up delivering in the drainage ditch outside of the maternity ward. I was told off by the same nurses that I was not serious with my work as a caretaker but they are the ones who rudely turned away the mother.

The international non-governmental organization that implemented and oversaw the running of the MWH for two years handed over responsibility for sustaining the facility to the Lundazi community in December 2018. Since that time, Agness noticed some differences in the overall functioning of the MWH. She shared:

From the time the [non-governmental organization] project phased out, I’m mostly alone at the mother’s shelter without a nurse or midwife. Hence I turn to handle situations alone when I have to take the mothers to the maternity ward. There is very little attention towards the mother’s shelter from the hospital staff most of the times. Even to have the administrative personnel just visiting the mother’s shelter takes a very long period of time between visits. Like this how can they know the challenges going on? My fellow community health workers feel I’m being paid a lot of money for being a caretaker but mostly I’m alone teaching the mothers.

Agness’ story highlights the central role caretakers play in contributing to the successful implementation and sustainability of MWH interventions. Agness is well positioned to be an agent of positive change by bringing health education to pregnant women. Her history living and working in rural Zambia gives her the unique ability to serve as caretaker of the MWH.

Agness talked about the use of traditional herbs by pregnant women to speed delivery and the difficulty she faces when educating mothers to avoid them. Reasons behind the use of traditional medicine during pregnancy among Zambian women include preparation for labor and delivery, prevention of childbirth complications, and prevention and/or treatment of anemia [3]. Family members and community leaders play an important role in influencing the use of traditional medicine during pregnancy in Zambia [3]. Agness’ story reminds us of the importance of considering traditional medicine use during pregnancy and the need to conduct qualitative research exploring the attitudes and rationale associated with the use of herbs during pregnancy.

Agness brought up the topic of disrespectful behavior from maternal healthcare providers. In recent years, a growing body of research has demonstrated that disrespect and abuse is prevalent around the globe including Zambia where researchers identified specific features in the environment of providers including personal experience, social norms, and organizational priorities that inhibit provision of respectful delivery care [4]. Agness’ story reminds us that research into barriers associated with the provision of respectful care during pregnancy and delivery is warranted.

Finally, her story demonstrates the need to ensure the long-term support and recognition of MWHs by communities as a benefit to pregnant women. Continued backing from local Ministry of Health officials after the inevitable phasing out of external funding will be crucial to the ongoing success of MWHs as an intervention to improve maternal-newborn health in Zambia and other low- and middle-income settings. An important lesson learned from Agness’ story is the need for future research to explore the sustainability of MWHs and long-term effectiveness of income generating activities by the community after the completion of externally funded implementing programs.
